# Impact of the COVID-19 pandemic on long-term sickness absences due to mental disorders in public servants: a retrospective observational study

**DOI:** 10.1186/s12889-025-22718-z

**Published:** 2025-04-22

**Authors:** Shinichi Iwasaki, Yasuhiko Deguchi, Shohei Okura, Kunio Maekubo, Ayaka Matsunaga, Koki Inoue

**Affiliations:** Department of Neuropsychiatry, Graduate School of Medicine, Osaka Metropolitan University, 1-4-3, Asahimachi, Abeno-ku, Osaka, 545-8585 Japan

**Keywords:** COVID-19 pandemic, Long-term sickness absence, Mental disorders, Public servants, Adjustment disorders, Interrupted time series analysis

## Abstract

**Background:**

The coronavirus disease 2019 (COVID-19) pandemic has profoundly impacted lives worldwide, influencing the incidence and severity of health problems. This may have affected the occurrence of workers’ sickness absences. This study aims to examine the incidence of long-term sickness absences due to mental disorders from 2009 to 2022 and the impact of the COVID-19 pandemic from the beginning of the COVID-19 pandemic (2020) to the end of the COVID-19 pandemic (2022).

**Methods:**

A retrospective observational design was employed. An anonymized record of public servants in City A with long-term sickness absences (≥ 90 days) from 2009 to 2022 was obtained. We defined 2009–2019 as the pre-COVID-19 pandemic period and 2020–2022 as the COVID-19 pandemic period. The influence of the COVID-19 pandemic on each disease category, classified using the ICD-10, was analyzed through interrupted time series analysis (ITSA) to evaluate changes in long-term sickness absences incidence before and during the COVID-19 pandemic period and the Cochran‒Armitage test to estimate trends in incidence rates over time.

**Results:**

Mental and behavioural disorders consistently demonstrated the highest incidence rates among all disease categories, with mood disorders being the most prevalent. Trends for all diseases analyzed did not change due to the COVID-19 pandemic. However, both ITSA and the Cochran‒Armitage test revealed increasing trends for depressive states and adjustment disorders throughout the study period.

**Conclusions:**

The COVID-19 pandemic did not influence the incidence of long-term sickness absences. However, depressive states and adjustment disorders exhibited an upward trend. This study underscores the need for tailored workplace mental health interventions to tackle the increasing stress-related illnesses, necessitating future research exploring the root causes.

**Supplementary Information:**

The online version contains supplementary material available at 10.1186/s12889-025-22718-z.

## Background

Long-term sickness absences (LTSA) have significant impacts on society across multiple dimensions, leading to diminished productivity, supplementary expenses related to sick pay and temporary replacements, financial strain from decreased income and heightened medical costs, as well as feelings of isolation, reduced self-esteem, and worsening mental health issues that affect an individual’s career and future [1]. The primary causes of LTSA are mental disorders and chronic physical ailments [2, 3], with mental health problems being a particularly significant factor [4]. The prevalence of LTSA due to mental disorders (LTSA-MDs) has been rising in several Western countries, with depression, anxiety, and stress-related disorders—referred to as common mental disorders—acting as the leading causes [5]. Additionally, LTSA-MDs tend to have longer durations compared to other causes [6]. Therefore, understanding trends in LTSA-MDs is essential for developing effective prevention and intervention strategies.

The coronavirus disease 2019 (COVID-19) pandemic has profoundly impacted lives worldwide and affected mental illness incidence and severity. Studies have reported increased COVID-19-related fear and anxiety, especially among females and younger adults in quarantine settings [7]. Fear of contracting COVID-19 poses a significant health risk by exacerbating anxiety, depression, and insomnia [8]. The pandemic has significantly increased the prevalence of major depressive and anxiety disorders, with economic stressors such as job losses and income reductions likely contributing to heightened stress levels and mental health issues [9, 10]. For many individuals, access to mental health services was restricted during the pandemic, potentially worsening pre-existing conditions or delaying timely treatment for new cases [11]. Remote work, which became widespread during the pandemic, has been associated with intensified psychological stress and isolation, leading to emotional, social, and psychological challenges. These challenges may contribute to depressive behaviours, reduced job satisfaction, and diminished quality of life [12, 13]. Few studies have explored the relationship between the COVID-19 pandemic and sickness absences. An increase in absences due to mental illness was reported during the early stages of the pandemic [14, 15]. Similarly, increased sickness absences due to respiratory and infectious diseases were observed during this period [15].

These factors may have influenced LTSA incidence caused by mental health conditions among workers. The pandemic’s impacts vary depending on national infection prevention measures and cultural contexts. In Japan, the Ministry of Health, Labour and Welfare has reported adverse effects on mental health caused by the pandemic [16]. The report highlights the effects of economic disruption, job loss, increased telework, income decline, and heightened household responsibilities [16]. These findings align with those of global studies. However, few studies have examined the effects of the COVID-19 pandemic on LTSA-MDs and there is limited empirical evidence examining its long-term effects on work participation. Most studies have focused on immediate psychological responses, while the potential delayed effects on workforce retention remain underexplored. Understanding the magnitude of LTSA is crucial for designing workplace mental health policies and interventions. This study will provide a comprehensive understanding of how the pandemic has shaped occupational mental health and long-term work participation.

We previously reported LTSA-MD incidence over the previous decade, as well as incidence trends [3]. Our findings indicated that LTSA caused by mental disorders was significantly more frequent than LTSA due to other physical diseases; mood disorders and major depressive disorders were the most commonly reported causes, maintaining high frequency between 2009 and 2018 among Japanese public servants. Furthermore, while LTSA caused by mood and major depressive disorders showed decreasing trends, the incidence of adjustment disorders increased during this period. However, this study only covered the pre-pandemic period (until 2018) and did not account for the pandemic’s impacts.

In this study, we aimed to examine the incidence of LTSA from 2009 to 2022, as well as the COVID-19 pandemic’s impact from 2020 to 2022, focusing on common mental disorders. Previous reports have linked the pandemic to various deteriorations in mental health, leading us to hypothesize that LTSA-MD incidence increased during the pandemic. By identifying trends in LTSA due to mental disorders, this study can inform workplace mental health strategies, early intervention programs, and policy adjustments aimed at reducing long-term work absences.

## Methods

### Study design and setting

A retrospective observational design was used to analyze existing data. We enrolled public servants working at either the municipal or ward office of City A in the Kinki region of Japan from 2009 to 2022. City A is one of the largest cities in Osaka Prefecture in the Kansai region of Japan.

### Participants and characteristics

Participants included those who began LTSA between 2009 and 2022. LTSA was defined as any sickness absence lasting ≥ 90 days. Participants who began LTSA between 2009 and 2022 were included from the year of their first LTSA episode. If a participant experienced multiple episodes, they were included for each year in which an episode occurred. The rate of occurrence of multiple LTSAs was 45.7%. Data on employees with one or more LTSA episodes during the study period were obtained anonymously from employment records using encrypted IDs. The dataset included the employees’ sex, age, and occupation (clerical, technical, or professional jobs, recorded from 2011 to 2022), reasons for sickness absences, and the start dates of LTSA episode(s). Clerical workers were defined as those performing administrative tasks related to construction, design, and management within the municipality. Technical workers undertook physically demanding roles, while professional workers included nurses, care workers, public health nurses, and childcare workers. Data on the total number of public servants, as well as workforce demographics such as sex, age, and occupation, were also collected for municipal and ward offices in City A between 2009 and 2022.

### The reasons for long-term sickness absences

Employers in City A are required to provide a medical certificate from a doctor for any sickness absence lasting ≥ 90 days. The medical certificate is prepared by the attending physician and includes one or more diagnoses. As the diagnoses are not listed based on International Classification of Diseases, Tenth Revision (ICD-10) code, researchers with ≥ 10 years of clinical experience converted each medical certificate into an ICD-10 code [17]. LTSA was classified as an LTSA-MD if the diagnosis corresponded to the F code in the ICD-10. When medical certificates listed multiple diagnoses, the primary diagnosis was determined based on the condition that necessitated the need for LTSA. If a secondary diagnosis was listed, it was only considered when the primary diagnosis was unclear. In such cases, clinical judgment was used to determine the main diagnosis. For example, if a patient had both a sleep disorder and a major depressive disorder, the diagnosis was classified as major depressive disorder. ICD-10 F3 includes mood disorders (e.g., major depressive and bipolar disorders), and F4 includes anxiety and stress-related disorders (e.g., generalized anxiety and adjustment disorders). Common mental disorders, including major depression (F32), depressive state (F329), and adjustment disorder (F43), were tallied separately from the total numbers of F3 and F4 individuals, as these are prevalent workplace disorders.

### Pre- and post-COVID-19 pandemic periods

Japan confirmed its first COVID-19 case in January 2020, enacted the Law on Special Measures against Novel Coronavirus in March 2020, and issued the first emergency declaration in April 2020. In May 2023, the government reclassified COVID-19 as a “category 5” infectious disease, aligning its treatment with that of common infectious diseases and easing restrictions on socioeconomic activities. For this study, we defined 2009–2019 as the pre-COVID-19 pandemic period and 2020–2022 as the COVID-19 pandemic period.

### Statistical analysis

The time variable was defined as yearly intervals from 2009 to 2022. The incidence rate was calculated as the number of LTSA incidents per 1,000 employees in City A in the same year. Disease categories with no recorded sickness absences during any year of the study period were excluded to reduce the effect of sparse data. Differences in LTSA rates across the study period were analyzed by sex, occupation, and age using a chi-square test. The Cochran‒Armitage test was used to estimate trends in incidence rates over time by diagnosis and sex for the entire study period.

The incident rate of LTSA was assessed by mean, variance, and Shapiro–Wilk test for estimating normal distribution. The rate of LTSA showed normal distribution and overdispersion. Therefore, we opted linear regression to model LTSA trends. An interrupted time series analysis (ITSA) was conducted to evaluate changes in LTSA incidence before and during the COVID-19 pandemic. ITSA assessed both level changes and trend changes in incidence during the pandemic compared with the pre-pandemic period. A continuous time variable was used to represent the observation period, capturing underlying trends in sick leave incidence independent of the pandemic. A COVID-19 dummy variable (with-COVID-19) distinguished between the pre-pandemic and pandemic periods. An interaction term between the time variable and the with-COVID-19 assessed whether the trend in LTSA incidence differed during the pandemic.

We specified a linear regression model with the time variable, with-COVID-19, and their interaction term to capture the pandemic’s effects on both the level and trend of sick leave incidence. The model used was as follows:

$$\displaylines{ Y\left(t \right) = {\beta _0} + {\beta _1}*Time + {\beta _2}\,with - COVID - 19 \cr + {\beta _3}*\left({Time \times with - COVID - 19} \right) + \varepsilon \left(t \right) \cr} $$

Here, Y(t) represents LTSA incidence at time t, β₀ represents baseline incidence pre-pandemic, β_1_ represents the pre-pandemic trend, β_2_ represents level changes during the pandemic, and β_3_ represents trend changes during the pandemic. ε(t) represents the error term.

Statistical analyzes were performed using SPSS version 29.0 (IBM Software Group, Chicago, IL) and Mac Statistical Analysis Ver. 3.0 (ESUMI, Tokyo, Japan). *P*-values < 0.05 were considered statistically significant.

## Results

### Participant characteristics

Table 1 presents the demographic characteristics of public servants, along with the number of LTSA episodes recorded from 2009 to 2022. The table summarises key data, including the total number of employees, sex distribution, occupational categories, age groups, and LTSA incidents. Throughout the study period, the workforce was predominantly male, though the proportion of female employees increased from 28.2% in 2009 to 40.8% in 2022. Clerical positions accounted for the largest workforce share, ranging from 53.6 to 61.6% across the years. The largest age group comprised those aged 40–49 years, representing approximately 29.7–35.3% of the sample.

**Table 1:** Characteristics of public servants and the number of long-term sickness absence episodes

	2009	2010	2011	2012	2013	2014	2015	2016	2017	2018	2019	2020	2021	2022	*p*
Total number of employee	21,394	20,708	21,376	21,392	20,404	19,513	18,679	18,520	19,085	18,641	18,492	19,503	20,119	20,448	
Male	15,358	14,746	14,840	14,705	13,881	12,951	12,212	11,970	12,156	11,872	11,761	12,002	12,131	12,106	***
	(71.8%)	(71.2%)	(69.4%)	(68.7%)	(68.0%)	(66.4%)	(65.4%)	(64.6%)	(63.7%)	(63.7%)	(63.6%)	(61.5%)	(60.3%)	(59.2%)	
Female	6,036	5,962	6,536	6,687	6,523	6,562	6,467	6,550	6,929	6,769	6,725	7,501	7,988	8,342	
	(28.2%)	(28.8%)	(30.6%)	(31.3%)	(32.0%)	(33.6%)	(34.6%)	(35.4%)	(36.3%)	(36.3%)	(36.4%)	(38.5%)	(39.7%)	(40.8%)	
Occupation															***
Clerical			11,452	11,783	11,390	11,462	11,257	11,358	11,598	11,445	11,399	11,738	12,236	12,534	
			(53.6%)	(55.1%)	(55.8%)	(58.7%)	(60.3%)	(61.3%)	(60.8%)	(61.4%)	(61.6%)	(60.2%)	(60.8%)	(61.3%)	
Technical			6459	6168	5570	4637	4043	3829	3774	3549	3432	3426	3341	3229	
			(30.2%)	(28.8%)	(27.3%)	(23.8%)	(21.6%)	(20.7%)	(19.8%)	(19.0%)	(18.6%)	(17.6%)	(16.6%)	(15.8%)	
Professional			3465	3441	3444	3414	3379	3333	3713	3647	3661	4339	4542	4685	
			(16.2%)	(16.1%)	(16.9%)	(17.5%)	(18.1%)	(18.0%)	(19.5%)	(19.6%)	(19.8%)	(22.2%)	(22.6%)	(22.9%)	
Age															***
-29	2007	1709	1817	1575	1507	1374	1243	1230	1275	1315	1394	1663	2025	2351	
	(9.4%)	(8.3%)	(8.5%)	(7.4%)	(7.4%)	(7.0%)	(6.7%)	(6.6%)	(6.7%)	(7.1%)	(7.5%)	(8.5%)	(10.1%)	(11.5%)	
30–39	6628	6377	6184	5877	5449	4967	4559	4187	4077	3518	3104	3101	2911	2796	
	(31.0%)	(30.8%)	(28.9%)	(27.5%)	(26.7%)	(25.5%)	(24.4%)	(22.6%)	(21.4%)	(18.9%)	(16.8%)	(15.9%)	(14.5%)	(13.7%)	
40–49	6766	6838	6827	6982	7072	6828	6546	6504	6735	6471	6374	6383	6331	6069	
	(31.6%)	(33.0%)	(31.9%)	(32.6%)	(34.7%)	(35.0%)	(35.0%)	(35.1%)	(35.3%)	(34.7%)	(34.5%)	(32.7%)	(31.5%)	(29.7%)	
50–59	5747	5597	5384	5364	4936	4889	4783	4938	5138	5439	5603	5978	6240	6359	
	(26.9%)	(27.0%)	(25.2%)	(25.1%)	(24.2%)	(25.1%)	(25.6%)	(26.7%)	(26.9%)	(29.2%)	(30.3%)	(30.7%)	(31.0%)	(31.1%)	
60-	246	187	1127	1594	1440	1455	1548	1661	1860	1898	2011	2378	2612	2873	
	(1.1%)	(0.9%)	(5.3%)	(7.5%)	(7.1%)	(7.5%)	(8.3%)	(9.0%)	(9.7%)	(10.2%)	(10.9%)	(12.2%)	(13.0%)	(14.1%)	
Number of LTSA incident	232	201	196	179	147	150	186	161	155	168	165	172	178	207	
(%)	(1.1%)	(1.0%)	(0.9%)	(0.8%)	(0.7%)	(0.8%)	(1.0%)	(0.9%)	(0.8%)	(0.9%)	(0.9%)	(0.9%)	(0.9%)	(1.0%)	
Male	174	143	139	125	103	101	126	111	108	116	107	127	118	127	
	(75.0%)	(71.1%)	(70.9%)	(69.8%)	(70.1%)	(67.3%)	(67.7%)	(68.9%)	(69.7%)	(69.0%)	(64.8%)	(73.8%)	(66.3%)	(61.4%)	
Female	58	58	57	54	44	49	60	50	47	52	58	45	60	80	
	(25.0%)	(28.9%)	(29.1%)	(30.2%)	(29.9%)	(32.7%)	(32.3%)	(31.1%)	(30.3%)	(31.0%)	(35.2%)	(26.2%)	(33.7%)	(38.6%)	
Occupation															**
Clerical	141	125	116	90	76	94	106	90	94	110	103	104	104	87	
	(60.8%)	(62.2%)	(59.2%)	(50.3%)	(51.7%)	(62.7%)	(57.0%)	(55.9%)	(60.6%)	(65.5%)	(62.4%)	(60.5%)	(58.4%)	(42.0%)	
Technical	68	54	56	53	54	37	56	52	47	42	43	44	40	45	
	(29.3%)	(26.9%)	(28.6%)	(29.6%)	(36.7%)	(24.7%)	(30.1%)	(32.3%)	(30.3%)	(25.0%)	(26.1%)	(25.6%)	(22.5%)	(21.7%)	
Professional	23	22	24	36	17	19	24	19	14	16	19	21	26	38	
	(9.9%)	(10.9%)	(12.2%)	(20.1%)	(11.6%)	(12.7%)	(12.9%)	(11.8%)	(9.0%)	(9.5%)	(11.5%)	(12.2%)	(14.6%)	(18.4%)	
Age															
-29	23	21	13	13	< 10	< 10	12	< 10	< 10	< 10	< 10	12	15	33	
	(9.9%)	(10.4%)	(6.6%)	(7.3%)			(6.5%)					(7.0%)	(8.4%)	(15.9%)	
30–39	52	65	53	39	41	28	41	34	30	34	29	20	21	24	
	(22.4%)	(32.3%)	(27.0%)	(21.8%)	(27.9%)	(18.7%)	(22.0%)	(21.1%)	(19.4%)	(20.2%)	(17.6%)	(11.6%)	(11.8%)	(11.6%)	
40–49	96	69	82	77	58	60	77	73	57	66	55	52	75	61	
	(41.4%)	(34.3%)	(41.8%)	(43.0%)	(39.5%)	(40.0%)	(41.4%)	(45.3%)	(36.8%)	(39.3%)	(33.3%)	(30.2%)	(42.1%)	(29.5%)	
50–59	60	46	45	47	38	52	54	50	56	55	73	75	61	76	
	(25.9%)	(22.9%)	(23.0%)	(26.3%)	(25.9%)	(34.7%)	(29.0%)	(31.1%)	(36.1%)	(32.7%)	(44.2%)	(43.6%)	(34.3%)	(36.7%)	
60-	< 10	< 10	< 10	< 10	< 10	< 10	< 10	< 10	< 10	< 10	< 10	13	< 10	13	
												(7.6%)		(6.3%)	

LTSA: Long-term sickness absences (> 90 days)

*: *p* < 0.05, ***: *p* < 0.001: Differences among workers with LTSA during the study period were assessed using the chi-square test

Data with less than 10 cases have been masked, as < 10, to ensure confidentiality

LTSA incidents varied annually, peaking at 232 in 2009, indicating a general downward trend in sickness absence episodes. Males accounted for 61.4–75.0% of LTSA incidents, whereas females accounted for 25.0–38.6%. The Cochran–‒Armitage test results for trend analysis revealed a statistically significant increasing trend (*p* < 0.01) in LTSA incidence.

### Frequency of occurrence classified by the disease causing LTSA

**Fig. 1:**
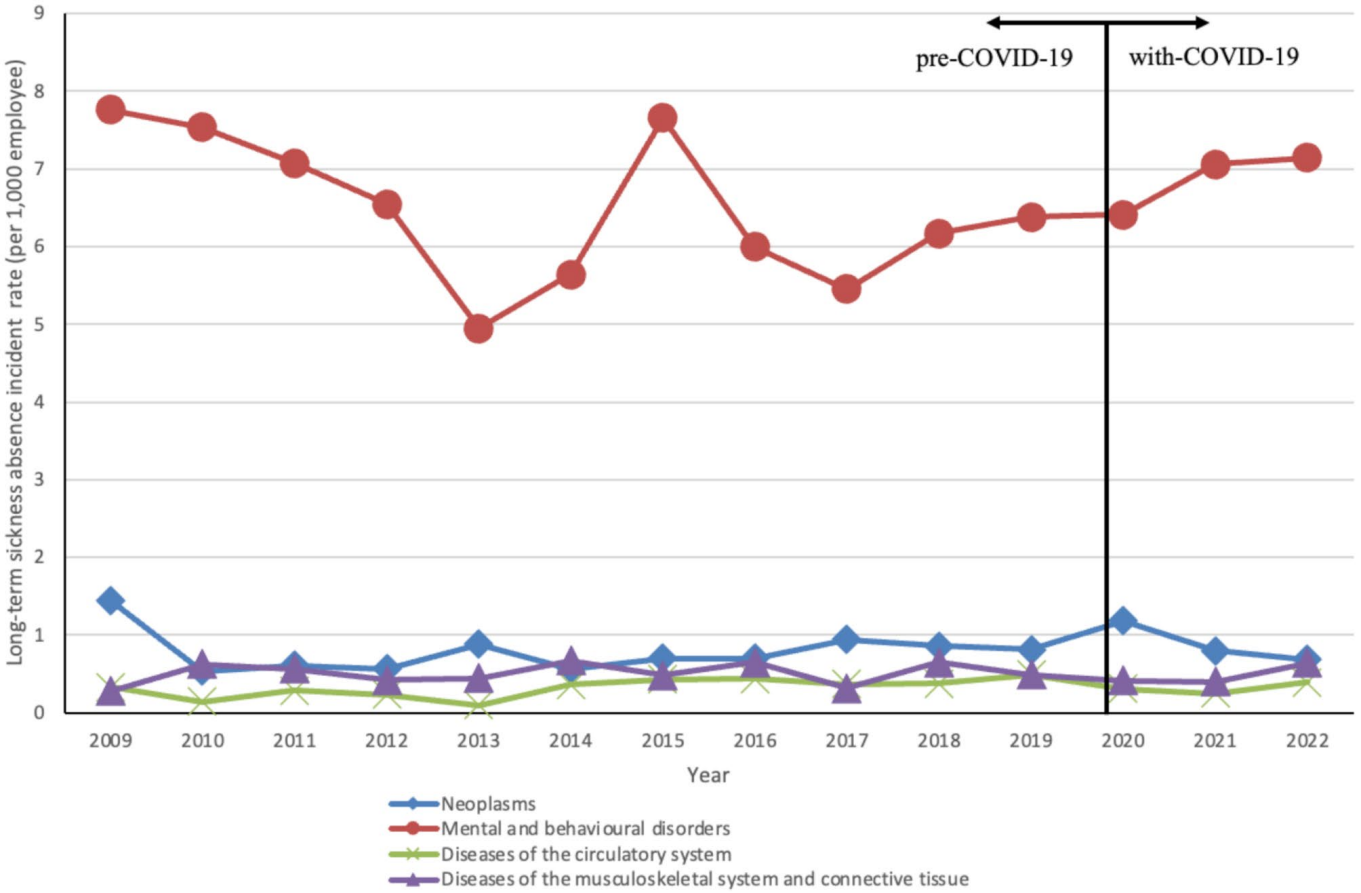
Annual incidence rates for each disease category causing long-term sickness absences from 2011–2022

Figure 1 shows trends in the frequency of LTSA caused by neoplasms, mental and behavioural disorders, circulatory system diseases, and musculoskeletal system and connective tissue diseases from 2009 to 2022. LTSA data for all diseases are presented in Additional file 1. LTSA associated with mental and behavioural disorders consistently formed the largest proportion of disease categories, although frequency fluctuated. Neoplasms, circulatory system diseases, and musculoskeletal and connective tissue diseases also demonstrated fluctuating frequencies, with no significant long-term trends detected.

### Frequency of occurrence classified by mental disorders causing long-term sickness absences

**Fig. 2:**
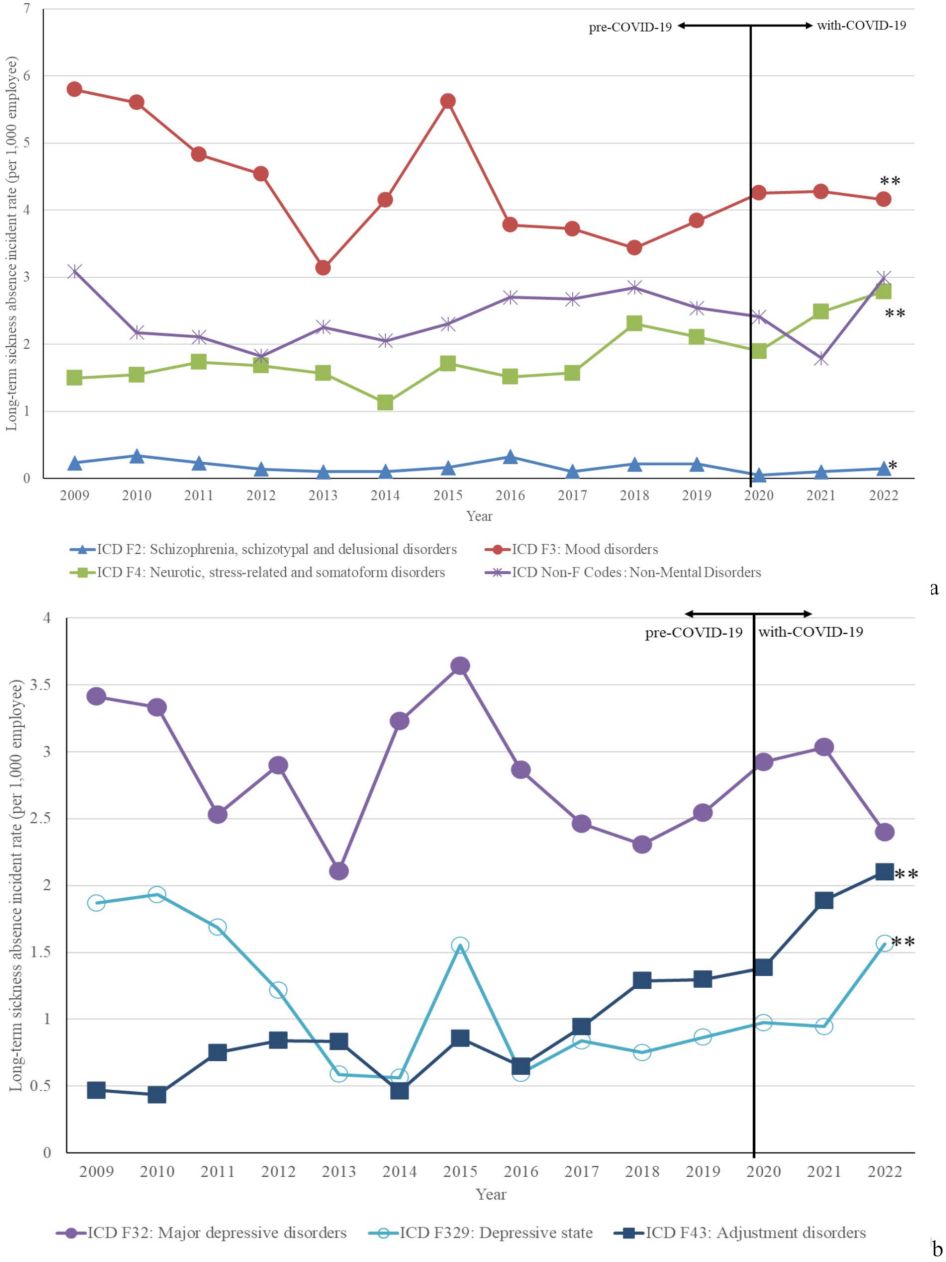
(**a**). Annual incidence rates for the most common LTSAs-causing mental disorder categories and subcategories (2011–2022). (**b**). Annual incidence rates for the most common mental disorder subcategories causing LTSAs (2009–2000) **: *p* < 0.01: analyzed by the Cochran‒Armitage test for trend. LTSA, long-term sickness absences

Figure 2a illustrates trends in LTSA frequency caused by specific mental disorders from 2009 to 2022. LTSA data for all psychiatric disorders are shown in Additional file 2. The Cochran‒Armitage test revealed a significant decrease in LTSA caused by schizophrenia, schizotypal, and delusional disorders (*p* < 0.05). LTSA due to mood disorders showed a significant overall decline from 2009 to 2022 (*p* < 0.01). Conversely, LTSA caused by neurotic, stress-related, and somatoform disorders demonstrated a significant increase over the study period (*p* < 0.01).

Figure 2b highlights trends in LTSA caused by common mental disorders from 2009 to 2022. This analysis focused on major depressive disorders, depressive states, and adjustment disorders. The Cochran‒Armitage test showed that LTSA caused by major depressive disorders fluctuated over time without a clear trend. LTSA due to depressive states exhibited a significant decreasing trend over time (*p* < 0.01). LTSA frequency caused by adjustment disorders increased significantly during the study period (*p* < 0.01).

### Interrupted time series analysis

The interrupted time series analysis of LTSA across major disease categories (Table 2) revealed varying trends before and during the COVID-19 pandemic. LTSA due to mental and behavioural disorders, neoplasms, and musculoskeletal disorders showed no significant trends during the overall study period or the pandemic. In contrast, circulatory system diseases exhibited a significant upward trend during the study period, though the pandemic did not significantly impact incidence trends. These findings suggest that the COVID-19 pandemic did not markedly alter the trajectory of LTSA incidence across the studied disease categories.

**Table 2:** Trend changes in long-term sickness absence incidence

		β	*p*	(95% CI)	*R* ^2^
Mental and behavioural disorders					0.30
	Time (year)	-0.69	0.101	(-0.32–0.03)	
	with-COVID-19	-2.32	0.52	(-20.33–10.94)	
	Time × with-COVID-19	3.02	0.41	(-0.80–1.82)	
Neoplasms					0.18
	Time (year)	-0.12	0.78	(-0.06–0.05)	
	with-COVID-19	5.05	0.21	(-1.98–8.05)	
	Time × with-COVID-19	-4.81	0.23	(-0.66–0.18)	
Diseases of the circulatory system					0.44
	Time (year)	0.93	0.02	(0.00–0.04)	
	with-COVID-19	-1.48	0.64	(-2.17–1.40)	
	Time × with-COVID-19	0.80	0.80	(-0.13–0.17)	
Diseases of the musculoskeletal system and connective tissue					0.15
	Time (year)	0.25	0.57	(-0.02–0.04)	
	with-COVID-19	-4.38	0.28	(-3.94–1.26)	
	Time × with-COVID-19	4.14	0.31	(-0.11–0.32)	

Data analyzed here was using interrupted time series analysis before and during the COVID-19 pandemic. COVID-19, Coronavirus disease

The analysis of LTSA caused by common mental disorders (Table 3) revealed distinct trends among diagnostic categories. Mood disorders showed a significant decline during the study period. Neurotic, stress-related, and somatoform disorders displayed a nonsignificant increase pre-pandemic, with a potential but nonsignificant rise during the pandemic. For major depressive disorders, neither significant pre-pandemic trends nor pandemic-related effects were observed. Depressive states significantly decreased over the study period (β = -0.97, *p* < 0.01, 95% CI: -0.20 to -0.03), with no significant pandemic-related changes. In contrast, adjustment disorders increased significantly during the study period (β = 0.57, *p* < 0.01, 95% CI: 0.03–0.11). Overall, while depressive states and adjustment disorders displayed significant trends, the COVID-19 pandemic itself did not significantly affect LTSA incidence for the most common mental disorders.

**Table 3:** Trend changes in long-term sickness absence incidence due to common mental disorders

		β	*p*	(95% CI)	*R* ^2^
Mood disorders					0.45
	Time (year)	-0.95	0.02	(-0.34 - -0.04)	
	with-COVID-19	-0.27	0.93	(-13.83–12.78)	
	Time × with-COVID-19	0.86	0.79	(-0.97–1.25)	
Neurotic, stress-related and somatoform disorders					0.72
	Time (year)	0.46	0.09	(-0.01–0.11)	
	with-COVID-19	-4.15	0.09	(-9.53–0.78)	
	Time × with-COVID-19	4.52	0.07	(-0.04–0.83)	
Major depressive disorders					0.22
	Time (year)	-0.59	0.17	(-0.16–0.03)	
	with-COVID-19	2.56	0.50	(-6.01–11.54)	
	Time × with-COVID-19	-2.20	0.56	(-0.93–0.54)	
Depressive state					0.51
	Time (year)	-0.97	0.01	(-0.20 - -0.03)	
	with-COVID-19	-3.54	0.25	(-11.49–3.32)	
	Time × with-COVID-19	4.28	0.17	(-0.21–1.03)	
Adjustment disorders					0.90
	Time (year)	0.57	0.00	(0.03–0.11)	
	with-COVID-19	-2.44	0.10	(-6.57–0.64)	
	Time × with-COVID-19	2.86	0.06	(-0.01–0.59)	

Data here was analyzed using interrupted time series analysis before and during the COVID-19 pandemic. COVID-19, Coronavirus disease 2019.

## Discussion

The study identified two key findings. First, the COVID-19 pandemic did not significantly alter trends in LTSA across various mental and physical diseases. Second, while certain mental disorder categories exhibited an overall declining trend during the study period, adjustment disorders showed a statistically significant linear increase. These findings underscore that the pandemic had limited effects on mental disorders overall, with adjustment disorders demonstrating a unique persistence and increase.

The first major finding indicates that the COVID-19 pandemic did not significantly influence LTSA trends across the analyzed disease categories, including neoplasms, diseases of the musculoskeletal system and connective tissue, diseases of the circulatory system, and diseases of the musculoskeletal system and connective tissue. The interrupted time-series analysis demonstrated no significant changes in trends before and during the pandemic in interaction terms between time and the COVID-19 period. These results suggest that, despite the considerable strain the pandemic placed on global health systems, its direct impact on LTSA incidence within these categories was not statistically significant. This contrasts with expectations of heightened mental health problems during the pandemic, suggesting that the trends in LTSA due to these conditions may have been offset by other factors. A study in the UK found elevated absenteeism rates for respiratory, infectious, and psychiatric disorders during the early pandemic but reported notable decreases in other diagnostic categories [15]. However, this study only encompassed the pandemic’s initial phases. Once infection waves subsided, absence rates returned to 2019 levels. Furthermore, the study included short absences (< 7 days), contrasting with this study’s focus on absences > 90 days. Based on these criteria, extended work absences due to COVID-19-related illnesses were less likely. Additionally, this study’s annual design did not reveal any changes in absence rates.

The COVID-19 pandemic has had varying effects on workers’ mental health. Numerous studies highlight its adverse effects on healthcare workers, including increased anxiety, depression, stress, burnout, acute stress disorder, post-traumatic stress disorder symptoms, and insomnia [18, 19]. However, while baseline psychological distress and physical symptoms were similar for healthcare and non-healthcare workers, indicators generally worsened for healthcare workers during the pandemic, whereas they remained stable or even improved among non-healthcare workers [20]. While mental health deterioration among healthcare workers has been widely reported, the pandemic’s effects on non-healthcare workers remain inconsistent.

On the other hand, the COVID-19 pandemic facilitated widespread adoption of remote work by organisations and employees [21, 22], yielding both positive and negative health outcomes. Many workers reported benefits of remote work, including better self-rated health, reduced commuting time, flexible working conditions, and lower risk of COVID-19 transmission [18, 23]. However, disadvantages such as technical challenges, blurred work-life boundaries, increased distractions, and social isolation have also been noted [24, 25]. Systematic reviews on remote work’s relationship with employee health remain inconclusive [26–29]. During the pandemic, public servants in City A adopted a hybrid work schedule, working from home on some days each week. Reports suggest hybrid work improves employee satisfaction and reduces turnover by one-third [30]. A study in China comparing commuters and teleworkers during the pandemic found no difference in mental health outcomes [31]. However, evidence on remote work’s effects on mental health remains inconsistent, with most studies originating from Western cultures. There is a notable lack of research examining these effects in Japan. Additionally, no studies have explored how remote work influenced LTSA trends related to mental disorders during the pandemic.

On the other hand, the COVID-19 pandemic led to both permanent and temporary job losses. While furloughs had no measurable impact on mental health, workplace downsizing was associated with a higher risk of anxiety. Furthermore, job loss significantly increased the risk of depression compared with stable employment [32]. However, this study focused on public servants—a highly stable occupational group in Japan—indicating that the impact of job instability was likely minimal. These findings suggest that the COVID-19 pandemic may not have substantially influenced leave of absence rates for nonhealthcare workers in stable occupations such as public servants. In summary, the effects of the COVID-19 pandemic on mental health, the expansion of remote work, and worsening employment conditions appear to have balanced each other, resulting in a net neutral effect on leave trends. However, the results for neuroticism (β = 4.52, 95% CI -0.04 to -0.83) and adjustment disorders (β = 2.86, 95% CI -0.01to -0.59) showed increasing trends, although not statistically significant, underscoring the need for further investigation.

The second major finding highlights an unstable decreasing trend in mood disorders, with only adjustment disorders exhibiting a linear increasing trend throughout the study period, as shown by interrupted time series analysis. This finding is significant because, even in the absence of substantial pandemic-related changes, the prevalence of LTSA due to stress-related illnesses continues to grow, whereas the prevalence of LTSA due to mood disorders is declining. The reduction in mood and depressive disorders may reflect the effectiveness of workplace mental health measures implemented before the pandemic. Although the overall prevalence of psychiatric disorders in Japan has been increasing [33], national policies such as the “Four Cares for Employees” (introduced in 2000), which were proposed by the Ministry of Health, Labor, and Welfare, and the “Stress Check System” (since 2015), which screens high-stress individuals, are national policies that screen for illnesses that are not well recognised and lead to treatment at medical institutions [34]. These initiatives to identify people with mental health problems at an early stage and link them to treatment at medical institutions mainly target mood disorders, particularly depression, which is the most common mental disorder in the workplace. These initiatives have been successful, and the number of employees on long-term leave owing to mood disorders seems to be decreasing. However, the number of individuals with known mental disorders and those with stress-related illnesses, including adjustment disorders—which are primarily addressed through environmental adjustments in the workplace rather than medical treatment—has risen. This suggests that the current measures are not adequately addressing these conditions. These findings underline the growing prevalence of mental health issues in society and highlight the need for targeted interventions specifically addressing stress-related and adjustment disorders.

The COVID-19 pandemic has not directly increased LTSA due to mental disorders but has contributed to a gradual increase in adjustment disorders. This aligns with previous research on healthcare challenges, such as human resource shortages and inadequate mental health support in Iranian government hospitals, during the pandemic [35]. Additionally, pre-hospital emergency services experienced high psychological distress among emergency responders due to lack of equipment and staff overload [36]. These studies suggest that stressors faced by healthcare workers and emergency responders could have long-term implications for occupational mental health. Sheikhbardsiri et al. highlighted the indirect effects of the pandemic on mental health for family caregivers of patients with COVID-19, emphasizing the indirect effects of the pandemic on mental health [37]. Future research should investigate the effect of occupational stress and caregiving burdens on delayed long-term absences.

These findings have significant implications for understanding the long-term effects of stress and mental health in the workplace, particularly among public employees. The observed decrease in LTSA due to mood disorders and the consistent increase in LTSA due to adjustment disorders highlight a broader trend that extends beyond the immediate context of the COVID-19 pandemic. This trend suggests that while current mental health support systems in organisational settings are effective for addressing mood disorders, they may be inadequate for managing stress-related conditions like adjustment disorders. Consequently, workplace policies should prioritise revising stress management and mental health interventions to address these emerging challenges better.

This study has several strengths. It leverages a long-term dataset spanning over a decade, enabling a robust analysis of trends both before and during the COVID-19 pandemic. The use of interrupted time series analysis provides a rigorous methodological framework for identifying changes in LTSA trends. Additionally, the focus on public servants offers valuable insights into a specific occupational group that may face unique stressors, contributing meaningful knowledge to occupational health research. However, the study has limitations. First, the use of anonymised records prevents exploration of individual-level factors or the direct psychological impact of COVID-19. Second, data on sickness absences under 90 days were not available, as public servants in City A are not required to provide medical certificates for such absences. The use of a 90-day threshold likely captured participants with more severe conditions compared to other studies, which may affect the comparability of results. Third, the focus on public servants in City A limits the generalizability of findings to other populations or occupational groups. Fourth, due to the relatively low number of LTSA cases and the relatively older participants, adjusting for age and sex would have reduced statistical power. Thus, we used crude models, which limit our findings. Finally, while it was assumed that the frequency and availability of remote work influenced sickness absence patterns, such information was not collected. Future research should aim to include more diverse populations, shorter LTSA durations, and a broader range of factors—including remote work arrangements—to enhance the applicability and depth of these findings.

## Conclusions

In conclusion, this study provides a detailed analysis of LTSA-related diseases during the COVID-19 pandemic and offers two key insights. First, the pandemic did not significantly alter LTSA trends across various disease categories, indicating that absenteeism patterns remained stable despite the unprecedented global health crisis. Second, the consistent increase in adjustment disorders and the decline in mood disorders highlight the critical need for ongoing mental health support in workplaces, particularly for managing chronic stress and related conditions. These findings emphasise the importance of tailoring workplace mental health interventions to address the growing burden of stress-related illnesses, which current systems may inadequately address. Longitudinal studies should examine whether the observed trends in LTSA persist or evolve post-pandemic. Given that adjustment disorders showed a steady increase, future research should explore whether this trend stabilizes, declines, or escalates in the coming years. Investigating these trends will help develop evidence-based strategies to mitigate the rising impact of mental health issues on workers.

## Electronic supplementary material

Below is the link to the electronic supplementary material.

**Additional file 1: Additional Figure 1:** Annual incidence rates of each disease in all categories that caused long-term absences from 2011 to 2022

**Additional file 2: Additional Figure 2:** Annual incidence rates of all mental disorder categories causing long-term sickness absences from 2011 to 2022

## Data Availability

All data generated or analysed in this study are included in the published article and its supplementary information files.

## Abbreviations

COVID-19: Coronavirus disease 2019
LTSA: Long-term sickness absences
LTSA-MDs: Long-term sickness absences due to mental disorders
ITSA: Interrupted time series analysis
