# Supplementary material for: Impact of the COVID-19 pandemic on long-term sickness absences due to mental disorders in public servants: a retrospective observational study
Source: BMC Public Health. 2025 Apr 22;25:1488. doi: 10.1186/s12889-025-22718-z (PMC12013149; doi:10.1186/s12889-025-22718-z)

Additional Figure 2. Annual incidence rates of all mental disorder categories causing long-term sickness absences from 2011 to 2022.


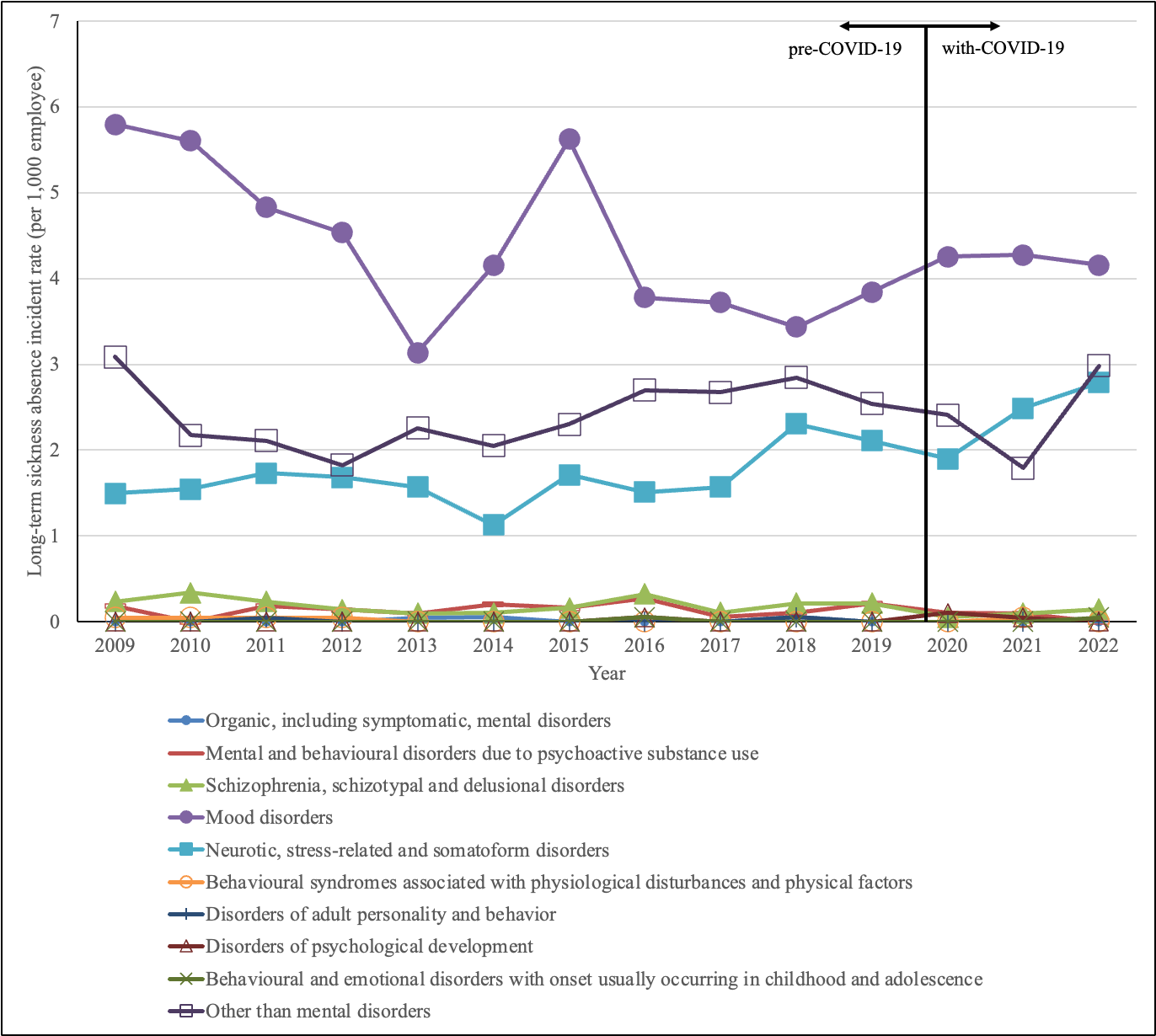

Supplement: Supplementary file 2 — Additional file 2: Additional Figure 2: Annual incidence rates of all mental disorder categories causing long-term sickness absences from 2011 to 2022 [file 12889_2025_22718_MOESM2_ESM.docx]
